# An unusual presentation of scrub typhus in a child: a case report

**DOI:** 10.1186/s12887-022-03139-y

**Published:** 2022-02-03

**Authors:** Jimba Jatsho

**Affiliations:** Department of Pediatrics, Phuentsholing Hospital, Ministry of Health, Phuentsholing, Bhutan

**Keywords:** Scrub typhus, *Rickettisia*, Children, Sensorineural hearing loss, Otalgia, Bhutan

## Abstract

**Background:**

Scrub Typhus (ST) is an acute, febrile zoonotic disease caused by the bacterium *Orientia tsutsugamushi* which is endemic to the Asia-Pacific region. Infected adults rarely present with sensorineural hearing loss and otalgia. Though few cases of pediatric cases are known to present with otalgia, no pediatric cases of sensorineural deafness complicating ST have been reported to date.

**Case presentation:**

A 5-year-old, previously healthy girl presented with a one-week history of high-grade intermittent fever, Headache and right ear pain with a recent onset of reduced hearing. She had a fever up to 39 °C, cervical lymphadenopathy, bilateral pleural effusion with diffuse infiltrations, and mild hepatosplenomegaly with no evidence of rash. Her initial examination was normal except for mildly enlarged tonsils. Unilateral right ear hearing loss was noted using Weber’s test. Evidence of progressive, mild anemia, and raised inflammatory markers was noted. Diagnosis of scrub typhus was confirmed by positive detection of *Orentia tsutsugamushi* IgM antibodies on rapid diagnostic test and the presence of chigger mite in the right external auditory canal on repeat examination. She responded dramatically to the empirical treatment of ceftriaxone and doxycycline. On follow-up, she did not have any residual hearing loss and her ear pain had resolved completely.

**Conclusion:**

Acute onset hearing loss or severe otalgia with or without findings should be an important diagnostic clue for suspecting scrub typhus in patients who present with a history of fever especially in endemic areas.

## Background

Scrub typhus (ST) is a mite-borne rickettsial zoonosis caused by *Orientia tsutsugamushi.* The trombiculid mites (genus *Leptotrombidium*) act as the vector and the disease is transmitted to humans through the bite from the infected larval stages (chiggers) of the mites [1]. ST presents as a simple febrile illness with eschar and rash but can sometimes present with a myriad of atypical and unusual manifestations [2]. Neurologically, patients usually present with severe headaches but can occasionally manifest as meningitis and meningoencephalitis, acute disseminated encephalomyelitis, and cranial nerve palsies [3–5]. In rare instances, patients infected with *Rickettsia rickettsii* can present with sensorineural hearing loss and otalgia [6–8]. No pediatric cases of sensorineural deafness complicating ST have been reported to date.

We report a case of ST in a child presenting with severe otalgia and unilateral sensorineural hearing loss, an uncommon presentation of this neglected tropical disease.

## Case presentation

A 5-year-old girl, a previously healthy child, presented with a one-week history of high-grade intermittent fever, headache, and right ear pain. She complained of paroxysmal right ear pain but without any history of ear discharge, tinnitus, dizziness, or seizures. She had a headache with nausea but no vomiting or loss of consciousness. This was associated with some abdominal distension and pain. Her bowel and urine output was normal, however, she had dysuria. The patient’s mother denied any history of insect or tick bite even though the child usually plays in the fields during the day. Both parents are farmers.

On the 7th day after the onset of illness, she complained of reduced hearing in her right ear without tinnitus, which gradually progressed to unilateral hearing loss.

Physical examination was notable for fever of 39 °C and cervical lymphadenopathy. There was no evidence of any rash or eschar. Examination of the throat revealed mildly enlarged tonsils without signs of inflammation. Initial otoscopic examination of the ears was unremarkable. Unilateral right ear sensorineural hearing loss was noted using Weber’s tuning fork test since audiometric tests weren’t available at our district hospital. She was mildly tachypneic but did not require oxygen. Bilateral pleural effusion was noted. Abdominal examination was notable for mild hepatosplenomegaly.

**Fig. 1:**
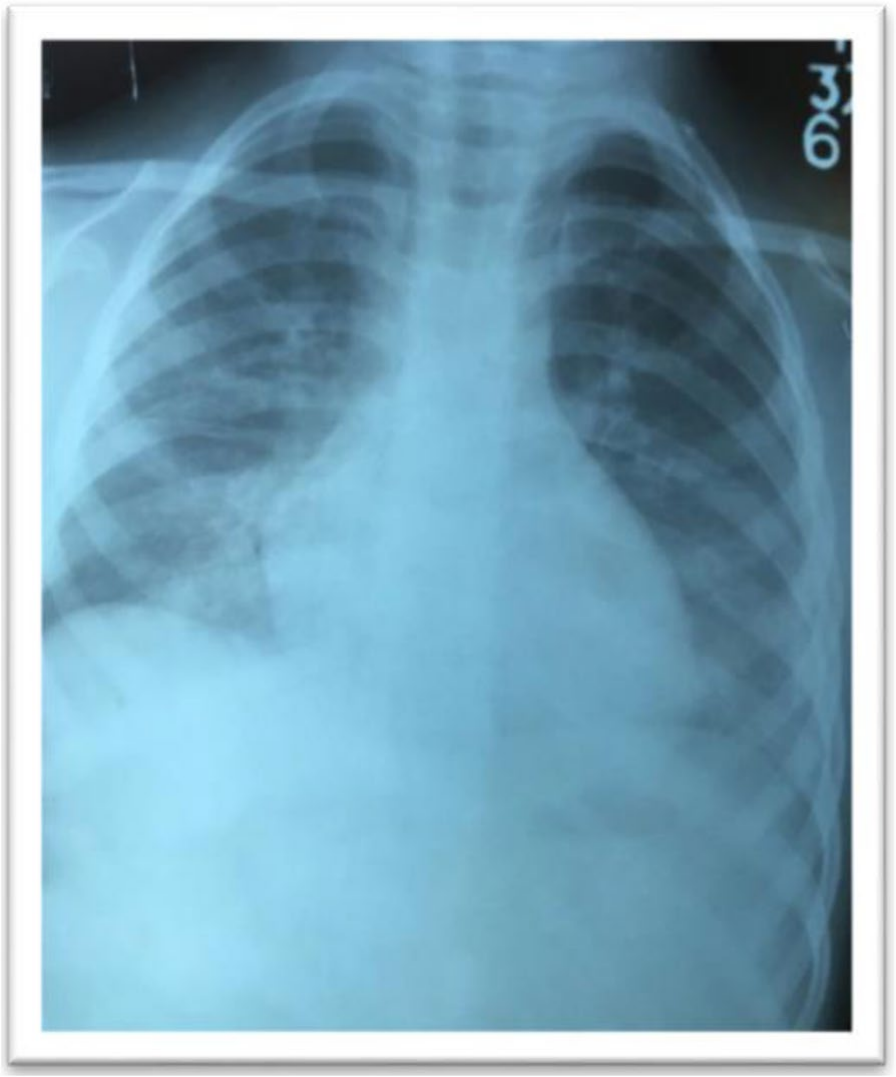
Chest X-ray showing minimal pleural effusion with bilateral diffuse infiltrates

Laboratory tests revealed thrombocytopenia (platelets 76,000 cells/mm^3^), mild anemia (hemoglobin 10.6 g/dL) and slightly raised neutrophils (70%). Subsequently, over the next few days, her platelet counts gradually decreased to a nadir of 47,000 cells/mm^3^. The C-reactive protein was 105 mg/dL with an increased erythrocyte sedimentation rate of 20 mm/hr. Urine revealed mild proteinuria. Chest X-ray showed bilateral pleural effusion with bilateral infiltrates (Fig. 1). An abdominal ultrasonogram confirmed mild hepatosplenomegaly. Rapid diagnostic tests (RDT) for leishmaniasis were negative but positive for dengue IgG. SD BIOLINE Tsutsugamushi RDT for scrub typhus was noted to be positive. SD BIOLINE Tsutsugamushi test is a rapid, qualitative, and differential test for the detection of *Orientia Tsutsugamushi* antibodies (IgG, IgM, IgA) in our setting [9].

**Fig. 2:**
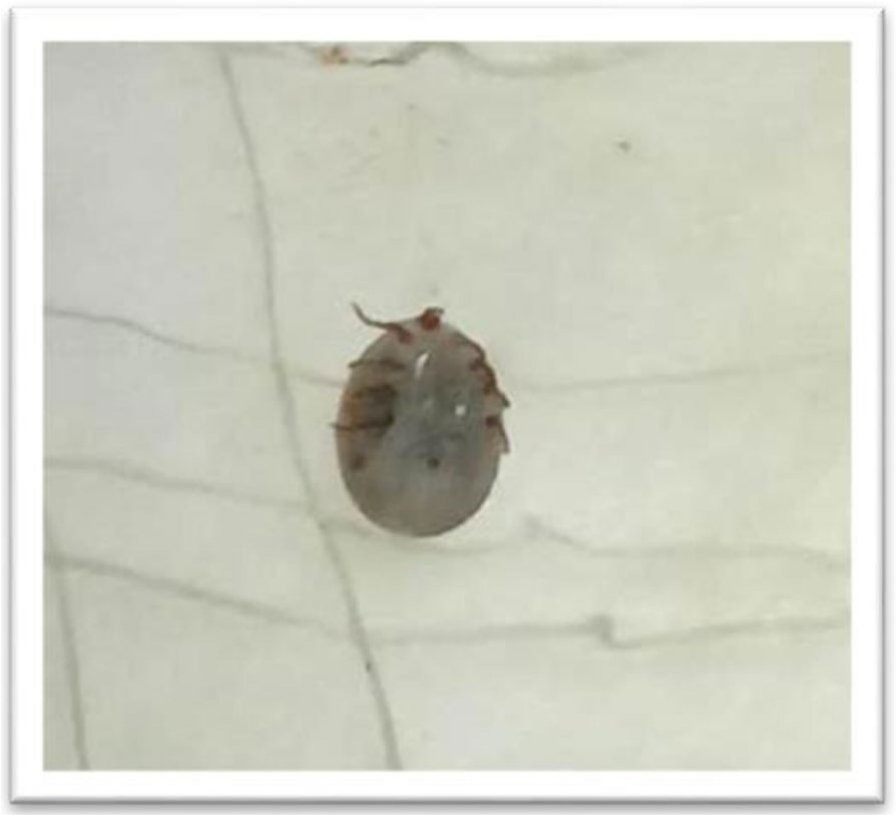
Trombiculid chigger mite removed from right external auditory canal

Empiric treatment was started with intravenous ceftriaxone and oral doxycycline. On the 6th day of admission, an otoscopic examination was repeated because of persistent mild ear pain and worsening hearing impairment. This revealed a chigger mite in the external auditory canal which was promptly removed (Fig. 2). However, there was no evidence of otitis media or inner ear abnormalities. Her pain settled soon after the removal of the mite and her fever resolved 2 days after the initiation of antibiotics.

At one-week follow-up, she did not have any residual hearing loss and the otalgia had resolved completely.

## Discussion and conclusions

Bhutan lies within the endemic area of scrub typhus historically referred to as the “tsutsugamushi triangle” [10]. ST cases have been reported since 2009 and became a notifiable disease of public health importance from 2010 [11] A recent serosurvey showed a high seroprevalence of scrub typhus and rickettsia in Bhutan [12]. In Bhutan, cases were likely underreported given the nonspecific clinical presentation, misdiagnosis by clinicians, and a lack of awareness of the disease [11].

Clinically, ST has a diverse clinical manifestation ranging from nonspecific febrile illness, rash, eschar, headache, myalgia, cough, generalized lymphadenopathy, nausea, vomiting, and abdominal pain to severe multiorgan dysfunction [13]. Fever and headache were the most common presenting symptoms [10]. The presence of an eschar, which is a typical painless necrotic skin lesion that may develop at the site of the bite, is pathognomonic. However, these are not present in several rickettsial diseases especially in children [14]. In Bhutan, common symptoms were nonspecific, and an eschar was noted by clinicians in only 7.4% of cases [12].

Central nervous system (CNS) manifestations are an uncommon and atypical feature of ST. Published case reports on CNS manifestations of ST in the pediatric population are limited. Among these, meningitis and meningoencephalitis are commonly reported with a subset of severe forms of patients presenting with altered sensorium and seizures [2]. In 2014, outbreaks of ST in a remote school in Bhutan lead to 2 deaths from meningoencephalitis [15].

Otalgia with sensorineural deafness is an unusual manifestation of ST. To date, no pediatric cases of sensorineural deafness complicating ST have been reported. Most of these cases described in the literature are in adult patients [6, 8, 16]. Premaratne et al. noted that hearing loss was a presenting symptom in 19% of patients with ST and affected up to one-third of the patients [8].

Our patient presented with nonspecific fever, headache, and associated sensorineural hearing loss, and severe otalgia. A sensory neural type of hearing loss has been reported in patients with rickettsial infections [7, 8, 17]. The actual mechanism of hearing loss in rickettsial diseases like ST is unclear. However, two possible pathological mechanisms have been postulated. Firstly, vasculitis-induced cochlear damage in the acute stage and secondly, an immune-mediated vasculitis in the vasa vasorum of the cochlear nerve [18, 19] have been proposed. In children, rickettsial infections are noted as a cause of secondary vasculitis [20].

In previous case reports in adults, otalgia usually appeared within the first week, followed by hearing loss and tinnitus in the second week after the onset of ST [6, 8]. Comparatively, in our patient, hearing loss appeared earlier within the first week of illness after the onset of otalgia. However, the reason for this early hearing loss in children is not known. Removal of the trigger of the inciting condition leads to remission of the vasculitis [20]. This corroborates the self-limiting course of hearing loss as in our patient.

Timely diagnosis and effective antibiotic treatment are effective in achieving good clinical outcomes [6]. However, in resource-limited settings like ours, mostly affordable RDTs allow rapid point-of-care testing and diagnosis. Serological tests like indirect immunofluorescence assays (IFAs) though sensitive are limited by the lack of standardization, variable cutoff titers for endemic regions, the requirement for paired sera, and high cost [21]. Other diagnostic methods include biopsy of eschar, culture, and polymerase chain reaction (PCR) including nucleic acid amplification (NAA). Early detection and diagnostic accuracy can be improved by combining NAA and IgM RDTs or Enzyme-Linked Immunosorbent Assays (ELISA) [22].

Clinicians working in endemic areas must consider ST and other rickettsial diseases in patients presenting with fever and sensorineural hearing loss or severe otalgia with or without otoscopic abnormalities.

## Data Availability

Data on patient and case details are available from the author on reasonable request.

## Abbreviations

ST: Scrub Typhus
CNS: Central nervous system
RDT: Rapid Diagnostic Test
NAA: Nucleic acid amplification
PCR: Polymerase chain reaction
IFA: Immunofluorescence assays
ELISA: Enzyme-Linked Immunosorbent Assays
